# Differences in Adolescents’ Alcohol Use and Smoking Behavior between Educational Tracks: Do Popularity Norms Matter?

**DOI:** 10.1007/s10964-021-01467-3

**Published:** 2021-07-07

**Authors:** M. Peeters, L. Laninga-Wijnen, R. Veenstra

**Affiliations:** 1 Interdisciplinary Social Science, Youth Studies, Utrecht University, Utrecht, The Netherlands; 2 Faculty of Social and Behavioural Science, Sociology, University of Groningen, Groningen, The Netherlands

**Keywords:** Popularity norms, Alcohol, Smoking, Adolescents

## Abstract

Explanations about differences in drinking and smoking rates between educational tracks have so far mainly focused on factors outside the classroom. The extent to which these behaviors are rewarded with popularity within a classroom—so called popularity norms—and their interaction with individual characteristics could explain the observed differences in risk behavior. 1860 adolescents (*M*_age_ = 13.04; 50% girls) from 81 different classrooms reported three times during one academic year about their own and their classmates behavior. Overall, in vocational tracks popularity norms for alcohol and smoking were more positive and predicted classroom differences in alcohol and smoking. Knowledge about classroom processes can advance the field in unraveling the functional aspects of risk behavior in adolescence. Preregistration: The hypotheses and the analytical plan of this study were preregistered under number #39136 (https://aspredicted.org/blind.php?x=gx77p6).

## Introduction

In several European countries, including the Netherlands, heavy alcohol use and smoking behavior in young adolescents is more common in vocational tracks compared with the higher educational tracks, the so-called social gradient of health risk behavior (Stevens et al.,^ 2018^; Mackenbach et al., 2015). Experimenting with drinking at an early age increases the risk for later problematic use (Grant & Dawson, 1997), and it is therefore crucial to understand the underlying reasons of early drinking behavior in young adolescents. Thus far, explanations for these educational differences have often been sought in the family context such as parenting behavior (De Looze et al., 2017) and family background (Hiscock et al., 2012) but overlooked the classroom context. Given that adolescents spend most of their time at school and in their classroom, it is likely that characteristics of this context contribute significantly to adolescents’ alcohol use and smoking (François et al., 2017). Particular popular peers in classrooms set a norm (popularity norm; Laninga‐Wijnen et al., 2017) given their heightened power and visibility in the peer group (Rambaran et al., 2013). However, the impact of classroom popularity norms on adolescents’ drinking and smoking behavior has not yet been investigated. Therefore, the aim of the current study is to examine the extent to which differences in alcohol and smoking behavior between educational tracks, can be explained by differences in alcohol and smoking popularity classroom norms. Additionally, the extent to which individual vulnerabilities (i.e. peer susceptibility and impulsivity) interact with classroom popularity norms will be investigated.

### Educational Differences in Risk Behavior

In the Netherlands, secondary schools have an early tracking system. In the last year of elementary school, students receive an advice based on test exams and the teachers’ opinion about the future academic achievements of the child. Adolescents are selected into pre-university education (“VWO”), higher general education (“HAVO”) or vocational track (“VMBO”). Within the vocational tracks students are selected for the theoretical (“VMBO-T”) or the practical track (“VMBO-K”). Adolescents with a lower family socioeconomic background are often overrepresented in the vocational tracks, and often, as young adults, acquire themselves a relatively lower socioeconomic position at the labor market (Lee & Byun, 2019). In the Netherlands, monitor studies indicate that in the vocational tracks, and in particular the practical tracks, drinking and smoking rates are much higher compared to other educational levels (Stevens et al., 2018). A similar pattern has been observed in other European countries (ESPAD, 2015).

According to evolutionary models of risk behavior (Ellis et al., 2012), the classroom context in vocational tracks could promote more risk behavior such as smoking and alcohol use compared with the classroom context in higher educational tracks. Early (social) maturation and a difference in future orientation could underlie the degree to which risk behavior is more prevalent in certain classroom contexts (Ellis et al., 2012; Frankenhuis & Del Giudice, 2012). Adolescents in vocational tracks often experience an earlier social maturation compared with their peers in higher academic tracks (Vermeulen et al., 2012). Adolescents with a lower socioeconomic family background also more likely have an early pubertal maturation compared with peers with a higher socioeconomic family background (Ellis & Essex, 2007). Early social and pubertal maturation foster behaviors with an adult status, such as drinking alcohol and smoking (Dijkstra et al., 2009; Ellis et al., 2012). Additionally, future prospects of adolescents in vocational tracks are characterized by more uncertainties with respect to income, education and employment than in higher educational tracks (Lee & Byun, 2019; Richter & Leppin, 2007). These uncertainties decrease the relevance of long-term strategies and favor short-term strategies (Frankenhuis & Del Giudice, 2012). A focus on short-term outcomes has been associated with more risk behavior such as alcohol use and smoking (Romer, 2010). The clustering of adolescents in vocational tracks with an relatively early social maturation, a focus on short term strategies combined with less bright future prospects may create a classroom environment in which risk behavior is more positively valued. In such classrooms, risk behaviors may be more likely endorsed by popular peers, leading to stronger positive alcohol and smoking popularity norms in vocational tracks compared with the higher educational tracks (hypothesis 1).

### Popularity Norms as Explanation for Educational Differences in Risk Behavior

The transition from childhood to early adolescence is characterized by a change in the behaviors that are accepted and rewarded with social status (Dijkstra et al., 2009). That is, rule breaking behaviors become more attractive and the willingness to engage in such behavior to enhance social status increases (LaFontana & Cillessen, 2010). This shift is often explained by the “maturity gap” referring to the developmental period in adolescence where adult like and rule breaking behaviors become more attractive as a means to close the gap between biological and social maturation (Moffitt, 1993; Dijkstra et al., 2015). These risk behaviors may therefore be seen in a positive light and may be related to popularity, particularly in contexts where the gap between biological maturity and social maturity is most prominent; in vocational tracks.

Descriptive norms are perceptions of what others typically do in a certain context (Borsari & Carey, 2001), such as within a classroom. Descriptive norms influence adolescents’ alcohol use and smoking behavior (Borsari & Carey, 2001; Veenstra et al., 2018). Popular adolescents have a strong impact on classroom norms; they decide who is accepted, who fits in, or who is rejected (Malamut et al., 2020; Veenstra et al., 2018), and their behaviors pose an important norm to others (Dijkstra & Gest, 2015). Not conforming to classroom norms can lead to social sanctions such as rejection or exclusion (e.g., social misfit theory, cf. Veenstra et al., 2018). According to the reputational salience hypothesis (Hartup, 1996), behaviors that are awarded with reputation and status, receive more value and become more salient than behaviors that do not reveal such association or are associated with peer rejection (Henry et al., 2000). The extent to which behaviors such as alcohol use and smoking are related to popularity in a certain classroom—also known as the popularity norm (Laninga‐Wijnen et al., 2017)—could be important in defining classroom differences in alcohol use and smoking behavior.

In line with this reasoning, prior work indicated that popular peers set a norm for the diffusion of risk attitudes in adolescent classrooms (Rambaran et al., 2013). Moreover, only risk preferences of popular adolescents (based on classroom nominations) were associated with the alcohol use of less popular best friends; this was not the case for risk preferences of less popular friends in relation to alcohol use of more popular friends (De Water et al., 2017). In addition, boys showed a higher willingness to drink when popular peers endorsed pro-alcohol norms, whereas their willingness to drink was substantially lower when popular peers displayed anti-alcohol norms (Teunissen et al., 2012). Adolescents changed their attitudes towards risk behavior stronger when exposed to a higher status peer than when exposed to a lower status peer (Teunissen et al., 2012). These findings suggest that particular the behavior of popular adolescents is influential on the risk attitudes and behaviors of other peers, and that popular peers set the norm within classrooms. In classrooms were drinking and smoking is rewarded with popularity, adolescents might be more inclined and motivated to conform to this norm and avoid exclusion. Classroom alcohol and smoking popularity norms could therefore predict higher drinking and smoking behavior (hypothesis 2).

### Individual Differences in Sensitivity to Classroom Influences

Susceptibility towards peer influence and impulsivity are two pivotal individual factors that may amplify the effect of peer influence on risk behaviors(Novak & Crawford, 2001; Teunissen et al., 2016; Meldrum et al., 2013). Not every individual may be equally sensitive to conform to the normative and socially rewarding behaviors in their classroom. Adolescents may vary in their susceptibility for peer influence and peer pressure and respond differently to peers’ beliefs, attitudes and behavior (Novak & Crawford, 2001; Telzer et al., 2020). Individual differences in susceptibility to peers’ substance use for instance predicts adolescents’ own drinking behavior (Teunissen et al., 2016). Adolescent boys (15–18 years) who were susceptible to peer influence and who had more friends who drank alcohol, drank more alcohol themselves. Such an effect was absent among the low susceptible adolescents. Susceptibility to peers is stronger when there is a need to fit in or receive social status (Meldrum et al., 2013). It is therefore expected that adolescents who are more susceptible to peer influence—and thus more likely adapt their own behavior to that of their peers would drink and smoke more in general (hypothesis 3) and this tendency may be amplified in classrooms with a strong alcohol and/or smoking popularity norm (hypothesis 4).

Impulsive adolescents have more difficulties in controlling their behavior and delaying rewards, which also has been observed in relation to drinking and smoking behavior (Fernie et al., 2013; Khuruna et al., 2013). Being impulsive may not only amplify the intrinsic rewards of these substances (e.g., relaxation, having fun; Hoeben et al., 2016) but also the socially rewarding effects (e.g., popularity status) of alcohol and smoking behavior (Meldrum et al., 2013). That is, good behavioral control skills as opposed to impulsivity, could assist adolescents in controlling their response and delay social rewards (Nelson et al., 2019; Romer et al., 2010), and as such withstand the influence of popularity classroom norms. The ability to inhibit or control the tendency to conform to the classroom norm could play an important role in explaining individual differences in susceptibility toward popularity classroom norms (Thomas & Marie McGloin, 2013). It was therefore expected that impulsive adolescents drink and smoke more (hypothesis 5; Fernie et al., 2013; Khuruna et al., 2013) and in particular when confronted with relatively strong alcohol and smoking popularity classroom norms (hypothesis 6).

## Present Study

Differences in alcohol and smoking behavior between educational tracks are a persistent problem, placing adolescents in the vocational tracks at greater risk for problematic risk behavior compared with their peers in higher educational tracks. Classroom differences in alcohol and smoking popularity norms could explain why problematic risk behavior is more pronounced in vocational educational tracks. A relatively earlier social maturation (Vermeulen et al., 2012) as well as a preference for short-term strategies typical in the lower educational tracks (Frankenhuis & Del Giudice, 2012) could lead to a stronger endorsement and attribution of popularity status to risk behavior (Ellis et al., 2012). Thus far, research on popularity norms in relation to alcohol use and smoking behavior is lacking. Therefore, the aim of the current study was to extend the current field of research in relation to popularity norms (Laninga-Wijnen et al., 2019) by examining the impact of alcohol and smoking popularity norms in relation to differences in alcohol use and smoking behavior between educational tracks. To evaluate individual behavior on a classroom level we applied multilevel techniques.

## Method

### **P**articipants and Procedure

Adolescents in this study participated in the Social Network Analysis of Risk behavior in Early adolescence (SNARE) project. This longitudinal study includes secondary school students, in their first and second grade, from two different schools in the north and middle of the Netherlands. The first cohort (2011–2012) included 7th and 8th graders; a year later, all new first year students (7th grade) were enrolled in a second cohort (2012–2013). In total, 1818 students (49.5% were girl) with a mean age of *M* age = 13.04 (SD = 0.70) from 81 classes were included for this study. Adolescents completed self-report and peer nominations during three regular assessments in one academic year. Almost all students completed each wave either during the regular assessment or within one month after the assessment, when they were absent. This resulted in the following sample size for the three waves that together covered one academic year: T1 (September, start of the academic year) N = 1818, T2 (October) N = 1814 (99.8% of the total sample) and T3 (December/January) N = 1806 (99.4% of the total sample). Sample size for the classroom level (popularity norms and educational level) was larger than sample size on the individual level due to use of peer nominations and classroom level information. For the outcome variable alcohol use and smoking more participants had missing information. Sample size for T1 was N = 1818, for T2 N = 1755 (96.5%), for T3 N = 1719 (94.5%). Attrition analyses revealed that those who dropped out were more likely in the higher educational tracks at T1 (t (1813) = −3.14, p < 0.01) and more likely to have smoked ever a cigarette at T1 (t (1738) = 2.01, p = 0.01). For alcohol use, impulsivity and peer susceptibility no differences were found. Missing data were handled using Full Information Maximum Likelihood (FIML).

The voluntary nature and the anonymity of the study was explained in a letter for parents as well as for adolescents themselves. Adolescents and parents could refuse participation by passive consent. In total, 40 students declined (2.2%). Ethical approval was provided by the Internal Ethical Board of the faculty of Social Sciences, Utrecht University.

### Measures

#### Alcohol use (T2–T3)

Alcohol use was assessed using a quantity-frequency measure (Sobell & Sobell, 1995). Adolescents indicated on how many days during the week (Monday–Thursday) and during the weekend (Friday-Sunday) in general they drink alcohol. In addition, adolescents indicated the average number of glasses consumed on such days, ranging from zero to 11 glasses or more. Drinking days and number of glasses were multiplied for the week and weekend separately and subsequently summed up together, resulting in an average alcohol consumption during the week (Peeters et al., 2017), with higher scores indicating more drinking. Cronbach’s alpha for these four items was 0.80. Factor analyses revealed a robust 1 factor component, resulting in an eigenvalue of 3.08, explaining 77% of the variance in the data.

#### Smoking (T2–T3)

Smoking behavior was assessed by asking adolescents to indicate the number of cigarettes they smoke on a single day with reference to the last month. Answer categories ranged from “never” to “more than 20 cigarettes a day”, with the other categories differentiating between irregular and daily smokers (i.e. “less than one cigarette a day/week” and “1–5”, “6–10”, “11–20”). Higher scores indicate more cigarette use during the day.

#### Peer susceptibility (T1)

Peer susceptibility was assessed with a newly developed scale in the SNARE study including six items. An example item is “Some youngsters do certain things that they normally would not do, because otherwise they do not longer belong to the group” and could be answered on a Likert type scale with categories ranging from “does not apply to me” to “does often apply to me”. Higher scores mean more perceived pressure and influence by friends. Cronbach’s alpha was 0.85 and a factor analyses revealed support for a robust one factor scale with a eigenvalue of 3.58 and 59% explained variance.

#### Impulsivity (T1)

Impulsivity was assessed with the Brief Self-Control Scale (Finkenauer et al., 2015). This scale has been used in previous studies using the SNARE sample, revealing good reliability (Franken et al., 2016, Cronbach’s alpha 0.77). Example items are “I am not able to work effectively toward long-term goals” and “I am easily discouraged”. Higher scores reflect higher levels of impulsivity. Cronbach’s alpha was 0.78.

#### Popularity norms (T2)

Smoking and alcohol popularity norms were assessed with peer nomination procedures within classrooms. Each adolescent was asked to indicate “who is most popular”, “who drinks alcohol” and “who smokes cigarettes”, referring to one of their classmates. For each student, the number of incoming nominations was calculated, which in turn was divided by the number of potential nominators within the classroom, resulting in a proportion score. Next, classroom popularity norms were calculated as the within-classroom correlation between students’ perceived popularity and perceived smoking and alcohol use, respectively. A Fisher’s r-to-z transformation was applied to ensure a normal distributions of these correlations. When there were no popularity nominations for alcohol/smoking (most likely indicative for non-use in the classroom), the correlation was set to zero which was the case for 24% of the classrooms for alcohol use, and 28% of the classrooms for smoking.

#### Education (T1)

In the Dutch school system, adolescents are selected in one of the four educational tracks; pre-university education (“VWO”), higher general education (“HAVO”) or lower vocational track (“VMBO”). Within the vocational tracks students are selected for the theoretical (“VMBO-T”) or the practical track (“VMBO-K”). Some students receive extra help in the practical track due to learning problems (“LWOO”). The classroom composition remains the same during the academic year. The two schools in the current study offered all educational tracks, though one school offered vocational and general education at different locations.

## Results

### Descriptive Statistics

Tables 1 and 2 include the descriptive statistic and Pearson correlations between study variables. Table 1 shows that alcohol use and smoking was more prevalent among the vocational tracks. Particularly, the practical vocational tracks (LWO and VMBO-K) revealed more drinking and smoking behavior compared with the other academic tracks. Positive correlations emerged for alcohol and smoking behavior at T2 and T3 were associated positively. Alcohol popularity norms were positively associated with smoking popularity norms. No significant associations with sex were found, with the exception for alcohol use at T2, indicating that boys drink more than girls.

**Table 1:** Descriptive statistics study variables total and separately for educational levels

	Mean (SD)	LWOO (*N* = 134)	VMBO-K (*N* = 203)	VMBO-T (*N* = 499)	HAVO (*N* = 104)	HAVO/VWO (*N* = 705)	VWO (*N* = 162)
Age	13.04 (0.70)						
Alcohol T2	0.46 (2.50)	0.88 (2.79)^a^	0.91 (3.34)^a^	0.54 (2.54)^ab^	0.51 (2.62)^ab^	0.29 (2.38)^b^	0.03 (0.16)^b^
Alcohol T3	0.46 (2.83)	0.53 (1.98)^ab^	0.88 (2.89)^a^	0.71 (4.14)^a^	0.38 (1.67)^ab^	0.25 (2.21)^b^	0.08 (0.43)^b^
Smoke T2	0.41 (2.31)	1.25 (4.40)^a^	1.01 (3.44)^ac^	0.31 (2.10)^b^	0.54 (2.07)^bc^	0.22 (1.65)^b^	0.03 (0.26)^b^
Smoke T3	0.54 (2.71)	0.90 (3.33)^abc^	1.16 (3.81)^a^	0.50 (2.55)^bc^	1.01 (3.68)^ac^	0.30 (2.09)^b^	0.39 (2.54)^bc^
Impulsivity	2.39 (0.60)	2.42 (0.64)^ab^	2.50 (0.65)^a^	2.39 (0.60)^b^	2.37 (0.70)^a^	2.35 (0.53)^b^	2.43 (0.67)^a^
Peer susceptibility	1.78 (0.68)	2.02 (0.74)^a^	2.00 (0.76)^a^	1.79 (0.70)^bc^	1.61 (0.62)^b^	1.74 (0.61)^bc^	1.59 (0.65)^b^

*LWOO* vocational extra help, *VMBO-K* vocational practical track, *VMBO-T* vocational theoretical track, *HAVO* higher general educational track, *VWO* pre-university track

Superscript indicates significant differences between educational level at *p* ≤ 0.05

**Table 2:** Pearson correlations between study variables separately for individual and classroom level variables

	1	2	3	4	5	6
Individual level (*N* = 1815)						
(1) Sex	1					
(2) Alcohol T2	0.06*	1				
(3) Alcohol T3	0.03	0.29**	1			
(4) Smoking T2	0.02	0.46**	0.22**	1		
(5) Smoking T3	0.04	0.31**	0.56**	0.32**	1	
(6) Impulsivity	−0.08**	0.08**	0.10**	0.10**	0.09**	1
(7) Peer pressure	0.04	0.07**	0.07**	0.08**	0.07**	0.47**
Classroom level (*N* = 81)	**1**	**2**				
(1) Alcohol pop_norm	1					
(2) Smoke pop_norm	0.72**	1				
(3) Education	−0.14	−0.24*				

^*^p < 0.05; **p < 0.01

To test the first hypothesis that alcohol and smoking popularity norms were more positive in vocational tracks compared with higher/pre-university tracks, a classroom-level ANOVA was conducted. Tables 3 and 4 present the means and differences between educational levels in alcohol and smoking popularity norms. Overall, no significant differences were found between education tracks for alcohol popularity norms *F*(75, 5) = 1.15, *p* = 0.34). For smoking popularity norms significant differences were observed *F*(75, 5) = 2.57 *p* = 0.03. For consistency reasons post hoc analyses were performed for both popularity norms. These analyses indicated that alcohol popularity norms were significantly more positive in the practical vocational tracks (VMBO-K) compared with the other educational tracks. For smoking popularity norms the two practical vocational tracks (LWOO and VMBO-K) revealed strong positive smoking popularity norms compared with the other educational tracks. Surprisingly, the higher general educational track (HAVO) revealed a relatively similar pattern as the practical vocational tracks. It should be noted, that differences need to be interpreted with caution as sample sizes of classes within each educational track varied and the total number of classrooms was relatively low for ANOVA comparison.

**Table 3:** Alcohol popularity norms for educational levels separately

	*N*	Mean	Sd
LWOO	10	0.29^ab^	0.42
VMBO-K	11	0.51^b^	0.39
VMBO-T	21	0.22^a^	0.42
HAVO	4	0.29^ab^	0.33
HAVO/VWO	29	0.22^a^	0.30
VWO	6	0.26^ab^	0.38

*LWOO* vocational extra help, *VMBO-K* vocational practical track, *VMBO-T* vocational theoretical track, *HAVO* higher general educational track, *VWO* pre-university track

Superscript indicates significant differences between educational level at *p* ≤ 0.05

**Table 4:** Smoking popularity norms for educational levels separately

	*N*	Mean	Sd
LWOO	10	0.32^a^	0.29
VMBO-K	11	0.26^a^	0.32
VMBO-T	21	0.03^b^	0.34
HAVO	4	0.24^a^	0.34
HAVO/VWO	29	0.07^b^	0.19
VWO	6	0.12^ab^	0.23

*LWOO* vocational extra help, *VMBO-K* vocational practical track, *VMBO-T* vocational theoretical track, *HAVO* higher general educational track, *VWO* pre-university track

Superscript indicates significant differences between educational level at *p* ≤ 0.05

### Multilevel Analyses of Alcohol Popularity Norms

A multilevel analyses using a negative binominal model to account for overdispersion (Peeters et al., 2012) was performed to test classroom level influences of alcohol popularity norm on individual drinking behavior (Table 5). In negative binominal models, explained variance and intraclass correlations have a different meaning compared to traditional multilevel model as a consequence of accounting for overdispersion in the data. For that reason they were not calculated, but instead variances for each model were presented (cf. Wang et al., 2010). First, a random intercept model was evaluated, only including alcohol use. Second, individual level main effects of peer susceptibility (*hypothesis 3*) and impulsivity (*hypothesis 5*) on alcohol use were investigated and classroom level variables were added to the model, starting with a model including only educational track and grade (model 1). At the individual level, peer susceptibility did not predict relative change in alcohol use. In contrast, impulsivity significantly predicted an increase in alcohol use (*b* = 1.02, *SE* = 0.31). This indicates that adolescents who were more impulsive revealed a relative increase in their drinking behavior. Educational track significantly predicted classroom differences in alcohol use, with higher drinking levels in the vocational tracks (*b* = −0.33, *SE* = 0.09, AIC = 9867, BIC = 9950). Alcohol popularity norms were added to the model (model 2) to observe whether popularity norms predicted higher classroom levels of alcohol use at T3 (*hypothesis 2*; *b* = 1.73, *SE* = 0.56, AIC = 9856, BIC = 9944). The predictive effect of educational track on alcohol use remained significant after adding popularity norms to the model (*b* = −0.25, *SE* = 0.09), though reduced in magnitude and the residual variance decreased (σ^2^ = 0.95 versus σ^2^ = 0.69). To examine possible cross-level interactions (*hypothesis 4 and 6*), first random slopes for peer susceptibility (*b* = 0.15, *SE* = 0.55) and for impulsivity (*b* = 0.46, *SE* = 0.52) were added simultaneously to the model. Slope variance coefficients were non-significant. In line with suggestions by LaHuis & Ferguson (2009) cross-level interactions were tested anyway, as a lack of power could have hindered us from detecting significant slope variance. Even though the model including both cross-level interactions resulted in a slightly better model fit (AIC = 2989 BIC = 3076) compared with a model with only random slopes (AIC = 3060, BIC = 3142) both cross level interactions were non-significant. Consequently, results did not support a cross level interaction for peer susceptibility (*b* = −1.16, *SE* = 1.00) or impulsivity (*b* = 0.17, *SE* = 1.25) with alcohol popularity norms. As such, the association between impulsivity and peer susceptibility and alcohol use did not vary as a function of classroom popularity norms (Table 6).

**Table 5:** Individual and classroom level effects of impulsivity, peer susceptibility, alcohol popularity norms, and their interaction on adolescents’ alcohol use at T3

	*b*	SE	95% CI	Deviance (AIC/BIC)
Random Intercept				1570/1586
Variance alcohol T3	3.76**	0.94	[1.34–6.18]	
Model 1				9867/9950
Variance alcohol T3	0.95**	0.19	[0.64–1.25]	
Alcohol T2	0.43**	0.10	[0.26–0.59]	
Sex	0.51	0.31	[0.04–1.01]	
Within-effect peer susceptibility	0.14	0.25	[−0.27–0.55]	
Within-effect impulsivity	1.02**	0.31	[0.51–1.52]	
Education	−0.33**	0.09	[−0.48–−0.18]	
Model 2				9856/9944
Variance alcohol T3	0.69**	0.20	[0.37–1.16]	
Alcohol T2	0.40**	0.11	[0.22–0.57]	
Sex	0.45	0.31	[−0.06–0.96]	
Within-effect peer susceptibility	0.20	0.26	[−0.23–0.63]	
Within-effect impulsivity	0.99**	0.31	[0.48–1.51]	
Education	−0.25**	0.09	[−0.41–−0.10]	
Popularity norms alcohol	1.73**	0.56	[0.81–2.65]	

Model 1 = model with individual main effects and education; Model 2 = model with alcohol popularity norms added

*p < 0.05; **p < 0.01

**Table 6:** Individual and classroom level effects of impulsivity, peer susceptibility, alcohol popularity norms, and their interaction on adolescents’ smoking behavior at T3

	*B*	SE	95% CI	Deviance (AIC/BIC)
Random intercept				1746/1763
Variance smoking T3	3.76**	0.94	[2.13–5.39]	
Model 1				9980/10063
Variance smoking T3	2.39**	0.51	[1.55–3.24]	
Smoke T2	0.47**	0.16	[0.21–0.73]	
Sex	0.94*	0.09	[0.28–1.60]	
Within effect peer susceptibility	0.45	0.26	[0.02–0.88]	
Within-effect impulsivity	0.63	0.46	[−0.13–1.39]	
Education	−0.36**	0.02	[−0.55–−0.17]	
Model 2				9978/10066
Variance smoking T3	2.39**	0.77	[1.14–3.65]	
Smoke T2	0.44*	0.18	[0.14–0.75]	
Sex	1.01*	0.40	[0.35–1.67]	
Within effect peer susceptibility	0.42	0.26	[−0.04–0.84]	
Within-effect impulsivity	0.63	0.46	[−0.13–1.39]	
Education	−0.31*	0.14	[−0.54–−0.07]	
Popularity norms smoke	1.96**	0.87	[0.29–3.17]	

Model 1 = model with individual main effects and education; Model 2 = model with alcohol popularity norms added

*p < 0.05; **p < 0.01

### Multilevel Analyses of Smoking Popularity Norms

A similar approach as with alcohol popularity norms was applied for smoking popularity norms. First, individual level factors were evaluated. Both peer susceptibility (*hypothesis 3*) and impulsivity (*hypothesis 5*) were non-significant predictors of individual smoking behavior. Educational track was a significant predictor for classroom level differences in smoking behavior (*b* = −0.36, *SE* = 0.02, AIC = 9980, BIC = 10,063). Being in the vocational tracks predicted more smoking behavior. Additionally, smoking popularity norms were added as predictor of class level smoking behavior. More positive norms at T2 predicted higher class level smoking behavior at T3 (*hypothesis 2*; *b* = 1.96, *SE* = 0.87, AIC = 9978, BIC = 10,066). After adding smoking popularity norms to the model the effect of educational level on smoking remained significant though decreased (*b* = −0.31, *SE* = 0.14). Residual variance in smoking behavior remained similar after adding smoking popularity norms (σ^2^ = 2.39 versus σ^2^ = 2.39). Adding random slopes to the model resulted in modeling issues that only could be solved with using a simpler estimation method, namely Maximum Likelihood using first order derivates (MLF) estimator instead of a MLR estimator. Random slopes for peer susceptibility (*b* = 0.13, *SE* = 0.20) and for impulsivity (*b* = 0.45, *SE* = 0.36) were none significant. In a next step, cross-level interactions were examined, however, model fit did not improve (random slopes model: AIC = 10,071, BIC = 10,175; cross level model: AIC = 10078, BIC = 10,199), and cross-level interactions were non-significant (susceptibility: *b* = 0.07, *SE* = 0.17); impulsivity: *b* = 0.04, *SE* = 0.14) suggesting no differences in the association between peer susceptibility (*hypothesis 4*), impulsivity (*hypothesis 6*) and smoking depending on the strength of smoking popularity norms within classrooms.

## Discussion

Differences in health risk behaviors between educational tracks, such as alcohol use and smoking have predominantly explained by factors outside the school context (De Looze et al., 2017; Hiscock et al., 2012. The findings of this study illustrate the importance of the classroom context as socializing environment for adolescents’ alcohol and drinking behavior. Alcohol and smoking popularity norms—which are more positive in vocational tracks—predict increase in drinking and smoking behavior among young adolescents. This suggest that adolescents in classrooms where alcohol use and smoking behavior is associated with popularity status, increase stronger in their drinking and smoking behavior compared with adolescents in classrooms in which such popularity norm is weaker.

### Educational Differences in Alcohol and Smoking Popularity Norms

In line with the first hypothesis, alcohol and smoking popularity norms were more positive in the lower educational tracks. This finding possibly may be explained by an earlier social maturation of adolescents in vocational tracks (Vermeulen et al., 2012), increasing the attractiveness of behavior with an adult status such as alcohol use and smoking. In addition, lower prospects with respect to income and job opportunities can favor short-term strategies that are immediately rewarding (Ellis et al., 2012; Frankenhuis & Del Giudice, 2012). Focusing on short-term outcomes has been associated with more risk behavior (Romer, 2010). The clustering of adolescents in vocational tracks with a similar future perspective as well as an relatively early social maturation could intensify the popularity status of risk behavior in these specific educational contexts.

Particularly for smoking and to a lesser extent for alcohol use, the prevalence in the higher general educational track resembles the prevalence in the vocational track (i.e. both are relatively high), prevalences also found in a national representative study of Dutch adolescents’ smoking and drinking behavior (Stevens et al., 2018). This finding illustrates that the reputational salience of risk behavior—in particular for smoking behavior—is not only typical for the vocational tracks, but could in a similar way affect adolescents’ smoking behavior in higher general educational tracks. Future research could illuminate additional factors such as school climate (cf. François et al., 2017) or the strength of the popularity hierarchy (e.g., accessibility of popularity status; Laninga-Wijnen et al., 2019) within a classroom. These factors could underlie classroom differences in reputational salience of risk behavior as well as differences in alcohol use and smoking behavior.

### Alcohol and Smoking Popularity Norms as Explanation for Educational Differences

Alcohol and smoking popularity norms played a partial role in explaining educational differences in alcohol and smoking behavior. Popularity norms for alcohol and smoking were more positive in lower vocational tracks, and were predictive of a relative increase in alcohol use and smoking behavior over time. These findings are in line with the reputational salience hypothesis (Hartup, 1996) indicating that behaviors that receive social status within a classroom become more salient.

Risky behaviors such as alcohol and smoking may serve different functions (e.g., increasing social status, avoid rejection, fitting in) within classrooms. The value which is assigned to risk behavior in terms of popularity status, could explain why in some classrooms risk behavior is more salient than in other classrooms. The reputational salience of risk behavior (Hartup, 1996), could affect individual behavior in two different ways both reflecting the need for social appraisal; that is, some adolescents may be triggered by the reward of being popular, and for that reason engage in alcohol and smoking behavior to improve their own social position. Others may fear rejection or not fitting in, and comply to the behavior of popular peers in class (Telzer et al., 2020), even if this behavior includes underage drinking and smoking behavior. From this latter perspective, alcohol use and smoking behavior could be behaviors deployed or used as means to secure one’s own position in the classroom.

Educational level remained a significant predictor of alcohol use and smoking behavior after considering the impact of popularity norms. The effect of educational level on classroom differences in alcohol and smoking behavior reduced for both risk behaviors after adding popularity norms to the model suggesting that alcohol and smoking popularity norms at least explain some of the classroom variance in alcohol use and smoking. A possible explanation for this partial effect could be the non-linear association between alcohol and smoking popularity norms and educational track. In the higher vocational tracks, popularity norms were more positive compared with the intermediate vocational track. Processes outside the classroom such as parental monitoring and parents’ socioeconomic position could be considered in future research and possible explain additional variation in drinking and smoking behavior between educational tracks. Parental monitoring for instance may not only affect classroom norms but also could affect educational attainment (Vermeulen et al., 2012). Parental socioeconomic background was found to be an important predictor of alcohol use and educational track in young adolescence, although unexplained effects on the educational level appear to predict an increase in alcohol use three years later (unpublished work, data can be requested from the first author). The results of the current study suggest that the classroom context may in addition to family socioeconomic background, play an important role in future drinking behavior of adolescents. Adolescents in vocational tracks, may more often than their peers in other educational tracks, be exposed to positive alcohol and smoking popularity norms. This exposure could increase the risk for an early onset of risk behavior and subsequently increasing the risk for later problematic use (Grant & Dawson, 1997). In other words, the classroom environment in vocational tracks could have an adverse effect on adolescents’ future prospects because of classroom processes such as the reputational salience of risk behavior.

### Individual Differences in Susceptibility to Alcohol and Smoking Popularity Norms

An additional aim of this study was to evaluate whether some students would be more open to the influence of popularity norms than others, based on their susceptibility to peer pressure and their impulsivity. Students with higher levels of impulsivity were more likely to drink but not more likely to smoke. Students’ susceptibility for peer pressure was unrelated to smoking and alcohol use. The differential role of impulsivity in relation to alcohol and smoking behavior is in line with a previous study revealing that impulsivity is a risk factor for the onset of alcohol and cannabis use at age 16 but not for smoking behavior (Peeters et al., 2017). This finding needs further investigation, though it is possible that different facets of impulsivity are related to different phases of smoking behavior (e.g., experimentation, initiation, addiction; Bloom et al., 2014). The finding of the current study illustrate that personality vulnerabilities such as impulsivity, impact adolescents’ drinking behavior regardless of the alcohol popularity norm within classrooms. This finding is in line with previous studies (Fernie et al., 2013; Khuruna et al., 2013), illustrating the important role of impulsivity as vulnerability marker of adolescents’ drinking behavior irrespective of the classroom context.

### Limitations

Some limitations may have affected the results and should be considered when interpreting the findings. First, although the sample size on the individual level was relatively large, on the classroom level, the sample size could be too small to detect cross-level interactions for instance. A Monte Carlo Simulation study indeed revealed that a larger sample size may be required to detect classroom differences (N = 180, power = 0.85, *p* < 0.05).

Second, in many classrooms (around 25%) adolescents nominated none of their peers as drinkers or smokers. As a result, the popularity norm was zero. Drinking and smoking rates are relatively low in this age group (Inchley et al., 2018), nevertheless, it is possible that some of these absent popularity norms were a result of insensitivity of the assessment method. For future research, it may be worthwhile to examine the sensitivity of popularity norms in identifying smokers and drinkers. In addition, although within classrooms some of these adolescents may not be exposed to popular peers who drink or smoke, on a school level, however these adolescents may be influenced by other popular peers at school or norms and values on the school level that affect smoking or drinking behavior. This norm outside the classroom context also affects adolescents in classroom with a strong/weak alcohol or smoking popularity norm. Therefore, peer norms outside the school context such as popularity norms in friendship networks or in sport clubs may affect the decision of adolescents to engage in drinking or smoking behavior as well. Future research could tackle this issue by including school level variables such as norms and school climate (Maes & Lievens, 2003).

Third, the analyses were controlled for grade but not for cohort. Since adolescents in the first cohort were both from 7th and 8th grade, and the second cohort only included 7th graders, the interrelationship between the two variables could lead to biased effects. In a model in which both variables were included, an opposite regression effects of the confounders was indeed found in comparison when observed separately (cf. Johnston et al., 2018). By controlling for grade analyses were indirectly also controlled for cohort, though it is possible that differences among 7^th^ graders between the two cohorts have affected the results. However, there were no reasons to assume that the influence of smoking and alcohol popularity norms on drinking and smoking varies between years. Last, adolescents were relatively young (mean age = 13.04) in this sample to observe regular drinking and smoking patterns. For alcohol use for instance, an onset before the age of 13 years is considered to be early (and problematic). To illustrate, in 2014 around 15% of the adolescents of 15 years, drunk alcohol on a weekly base (Inchley et al., 2018). Although this sample of young adolescents has the ideal age to identify experimentation with substance use and development of norms, future research could include adolescents from higher grades as well. Reputational salience of alcohol and smoking may affect drinking and smoking behavior of adolescents differently as these behaviors become more prevalent.

### Implications

The findings of this study emphasize to consider the functionality of behavior within the context (Ellis et al., 2012). More positive alcohol and smoking popularity norms in vocational tracks may increase feelings of not fitting in or being rejected when not conforming to these popularity norms. Understanding of the reasons behind involvement in risk behavior such as alcohol use and smoking may support the development of successful approaches to reduce educational disparities in health risk behaviors (Yeager et al., 2018). Promising interventions that integrate the social network and select popular peers to act as supporters of healthy behavior such as ASSIST (A Stop Smoking in School Trial; Campbell et al., 2008; also applied in physical health; Van Woudenberg et al., 2019) could support schools in decreasing alcohol and smoking behavior among adolescents.

## Conclusion

Alcohol and smoking popularity norms within the classroom play an important role in adolescents’ alcohol and drinking behavior and partly explain differences in health risk behaviors between educational tracks. The enhanced reputational salience of alcohol and smoking within the classrooms, possibly creates an environment in which it is more difficult for adolescents to withstand the urge to smoke or drink alcohol. Adolescents in vocational tracks may more often than their peers in higher educational tracks, be exposed to the socially rewarding consequences of drinking and smoking behavior, and as such the functionality of this behavior may have a different meaning in lower vocational tracks. The classroom environment and associated peer dynamics should therefore be considered as an important socializing context in relation to differences in adolescents’ risk behavior between educational tracks.

## References

[CR1] Bloom EL, Matsko SV, Cimino CR (2014). The relationship between cigarette smoking and impulsivity: A review of personality, behavioral, and neurobiological assessment. Addiction Research & Theory.

[CR2] Borsari B, Carey KB (2001). Peer influences on college drinking: A review of the research. Journal of substance abuse.

[CR3] Campbell R, Starkey F, Holliday J, Audrey S, Bloor M, Parry-Langdon N, Moore L (2008). An informal school-based peer-led intervention for smoking prevention in adolescence (ASSIST): a cluster randomised trial. The Lancet.

[CR4] Dijkstra JK, Gest SD (2015). Peer norm salience for academic achievement, prosocial behavior, and bullying: implications for adolescent school experiences. The Journal of Early Adolescence.

[CR5] Dijkstra JK, Lindenberg S, Verhulst FC, Ormel J, Veenstra R (2009). The relation between popularity and aggressive, destructive, and norm-breaking behaviors: moderating effects of athletic abilities, physical attractiveness, and prosociality. Journal of Research on Adolescence.

[CR6] Dijkstra JK, Kretschmer T, Pattiselanno K, Franken A, Harakeh Z, Vollebergh W, Veenstra R (2015). Explaining adolescents’ delinquency and substance use: a test of the maturity gap. Journal of Research on Crime and Delinquency.

[CR7] Ellis BJ, Essex MJ (2007). Family environments, adrenarche, and sexual maturation: a longitudinal test of a life history model. Child Development.

[CR8] Ellis BJ, Del Giudice M, Dishion TJ, Figueredo AJ, Gray P, Griskevicius V, Wilson DS (2012). The evolutionary basis of risky adolescent behavior: implications for science, policy, and practice. Developmental Psychology.

[CR9] Fernie G, Peeters M, Gullo MJ, Christiansen P, Cole JC, Sumnall H, Field M (2013). Multiple behavioural impulsivity tasks predict prospective alcohol involvement in adolescents. Addiction.

[CR10] Finkenauer C, Buyukcan-Tetik A, Baumeister RF, Schoemaker K, Bartels M, Vohs KD (2015). Out of control: Identifying the role of self-control strength in family violence. Current Directions in Psychological Science.

[CR11] François A, Lindstrom Johnson S, Waasdorp TE, Parker EM, Bradshaw CP (2017). Associations between adolescents’ perceptions of alcohol norms and alcohol behaviors: incorporating within-school variability. American Journal of Health Education.

[CR12] Franken A, Moffitt TE, Steglich CE, Dijkstra JK, Harakeh Z, Vollebergh WA (2016). The role of self-control and early adolescents' friendships in the development of externalizing behavior: The SNARE study. Journal of youth and adolescence.

[CR13] ESPAD Report. (2015). Results from the European School Survey Project on alcohol and other drugs.

[CR14] Frankenhuis WE, Del Giudice M (2012). When do adaptive developmental mechanisms yield maladaptive outcomes?. Developmental Psychology.

[CR15] Grant BF, Dawson DA (1997). Age at onset of alcohol use and its association with DSM-IV alcohol abuse and dependence: results from the National Longitudinal Alcohol Epidemiologic Survey. Journal of Substance Abuse.

[CR16] Hartup WW (1996). The company they keep: friendships and their developmental significance. Child Development.

[CR17] Henry D, Guerra N, Huesmann R, Tolan P, VanAcker R, Eron L (2000). Normative influences on aggression in urban elementary school classrooms. American Journal of Community Psychology.

[CR18] Hiscock R, Bauld L, Amos A, Fidler JA, Munafò M (2012). Socioeconomic status and smoking: a review. Annals of the New York Academy of Sciences.

[CR19] Hoeben EM, Meldrum RC, Walker DA, Young JT (2016). The role of peer delinquency and unstructured socializing in explaining delinquency and substance use: a state-of-the-art review. Journal of Criminal Justice.

[CR20] Inchley, J. C., Currie, D. B., Vieno, A., Torsheim, T., Ferreira-Borges, C., Weber, M.,… & Breda, J. (2018). *Adolescent alcohol-related behaviours: trends and inequalities in the WHO European Region, 2002–2014*. WHO Regional Office for Europe.

[CR21] Johnston R, Jones K, Manley D (2018). Confounding and collinearity in regression analysis: a cautionary tale and an alternative procedure, illustrated by studies of British voting behaviour. Quality & Quantity.

[CR22] Khurana A, Romer D, Betancourt LM, Brodsky NL, Giannetta JM, Hurt H (2013). Working memory ability predicts trajectories of early alcohol use in adolescents: the mediational role of impulsivity. Addiction.

[CR23] LaFontana KM, Cillessen AH (2010). Developmental changes in the priority of perceived status in childhood and adolescence. Social Development.

[CR24] LaHuis DM, Ferguson MW (2009). The accuracy of significance tests for slope variance components in multilevel random coefficient models. Organizational Research Methods.

[CR25] Laninga-Wijnen L, Harakeh Z, Garandeau C, Dijkstra JK, Veenstra R, Vollebergh WAM (2019). Classroom popularity hierarchy predicts prosocial and aggressive popularity norms across the school year. Child Development.

[CR26] Laninga‐Wijnen L, Harakeh Z, Steglich C, Dijkstra JK, Veenstra R, Vollebergh W (2017). The norms of popular peers moderate friendship dynamics of adolescent aggression. Child Development.

[CR27] Lee B, Byun SY (2019). Socioeconomic status, vocational aspirations, school tracks, and occupational attainment in South Korea. Journal of Youth and Adolescence.

[CR28] de Looze ME, van Dorsselaer SA, Monshouwer K, Vollebergh WA (2017). Trends in adolescent alcohol use in the Netherlands, 1992–2015: differences across sociodemographic groups and links with strict parental rule-setting. International Journal of Drug Policy.

[CR29] Mackenbach JP, Kulhánová I, Bopp M, Borrell C, Deboosere P, Kovács K, De Gelder R (2015). Inequalities in alcohol-related mortality in 17 European countries: a retrospective analysis of mortality registers. PLoS medicine.

[CR30] Maes L, Lievens J (2003). Can the school make a difference? A multilevel analysis of adolescent risk and health behaviour. Social Science & Medicine.

[CR31] Malamut, S. T., van den Berg, Y. H., Lansu, T. A., & Cillessen, A. H. (2020). Bidirectional associations between popularity, popularity goal, and aggression, alcohol use and prosocial behaviors in adolescence: a 3-year prospective longitudinal study. *Journal of Youth and Adolescence*, 1–16. 10.1007/s10964-020-01308-9. 10.1007/s10964-020-01308-9 PMC7875842 32865706

[CR32] Meldrum RC, Miller HV, Flexon JL (2013). Susceptibility to peer influence, self‐control, and delinquency. Sociological Inquiry.

[CR33] Moffitt TE (1993). Adolescence-limited and life-course-persistent antisocial behavior: a developmental taxonomy. Psychological Review.

[CR34] Nelson TD, Nelson JM, Mason WA, Tomaso CC, Kozikowski CB, Espy KA (2019). Executive control and adolescent health: toward a conceptual framework. Adolescent Research Review.

[CR35] Novak KB, Crawford LA (2001). Perceived drinking norms, attention to social comparison information, and alcohol use among college students. Journal of Alcohol and Drug Education.

[CR36] Peeters M, Oldehinkel T, Vollebergh W (2017). Behavioral control and reward sensitivity in adolescents’ risk taking behavior: a longitudinal TRAILS study. Frontiers in Psychology.

[CR37] Peeters M, Wiers RW, Monshouwer K, van de Schoot R, Janssen T, Vollebergh WA (2012). Automatic processes in at‐risk adolescents: the role of alcohol‐approach tendencies and response inhibition in drinking behavior. Addiction.

[CR38] Rambaran AJ, Dijkstra JK, Stark TH (2013). Status‐based influence processes: the role of norm salience in contagion of adolescent risk attitudes. Journal of Research on Adolescence.

[CR39] Richter M, Leppin A (2007). Trends in socio-economic differences in tobacco smoking among German schoolchildren, 1994–2002. European Journal of Public Health.

[CR40] Romer D (2010). Adolescent risk taking, impulsivity, and brain development: Implications for prevention. Developmental Psychobiology: The Journal of the International Society for Developmental Psychobiology.

[CR41] Romer D, Duckworth AL, Sznitman S, Park S (2010). Can adolescents learn self-control? Delay of gratification in the development of control over risk taking. Prevention Science.

[CR42] Sobell, L. C., & Sobell, M. B. (1995). Alcohol consumption measures. In *Assessing alcohol problems: a guide for clinicians and researchers*, 2 (pp. 75–99), U.S. Department of Health and Human Services, Washington, D.C.

[CR43] Stevens, G. W. J. M., van Dorsselaer, S., Boer, M., de Roos, S., Duinhof, E. L., ter Bogt, T. F. M., ... & de Looze, M. (2018). HBSC 2017. Gezondheid en welzijn van jongeren in Nederland. Utrecht University.

[CR44] Telzer, E. H., Jorgensen, N. A., Prinstein, M. J., & Lindquist, K. A. (2020). Neurobiological sensitivity to social rewards and punishments moderates link between peer norms and adolescent risk taking. *Child Development*. 10.1111/cdev.13466. 10.1111/cdev.13466 PMC8340982 33030267

[CR45] Teunissen HA, Spijkerman R, Prinstein MJ, Cohen GL, Engels RC, Scholte RH (2012). Adolescents’ conformity to their peers’ pro‐alcohol and anti‐alcohol norms: The power of popularity.. Alcoholism: Clinical and experimental research.

[CR46] Teunissen HA, Kuntsche E, Scholte RH, Spijkerman R, Prinstein MJ, Engels RC (2016). Friends’ drinking norms and male adolescents’ alcohol consumption: The moderating role of performance-based peer influence susceptibility. Journal of Adolescence.

[CR47] Thomas KJ, Marie McGloin J (2013). A dual‐systems approach for understanding differential susceptibility to processes of peer influence. Criminology.

[CR48] Veenstra, R., Dijkstra, J. K., & Kreager, D. A. (2018). Pathways, networks, and norms: a sociological perspective on peer research. In W. M. Bukowski, B. Laursen, & K. H. Rubin (Eds.), *Handbook of peer interactions, relationships, and groups* (pp. 45–63). The Guilford Press.

[CR49] Vermeulen-Smit E, Ter Bogt TF, Verdurmen JE, Van Dorsselaer SA, Vollebergh WA (2012). The role of education, parents and peers in adolescent heavy episodic drinking. Drugs: Education, Prevention and Policy.

[CR50] Wang M, Liu S, Zhan Y, Shi J (2010). Daily work–family conflict and alcohol use: testing the cross-level moderation effects of peer drinking norms and social support. Journal of Applied Psychology.

[CR51] De Water E, Burk WJ, Cillessen AH, Scheres A (2017). Substance use and decision‐making in adolescent best friendship dyads: The role of popularity. Social Development.

[CR52] Van Woudenberg TJ, Simoski B, de Mello Araújo EF, Bevelander KE, Burk WJ, Smit CR, Buijzen M (2019). Identifying influence agents that promote physical activity through the simulation of social network interventions: agent-based modeling study. Journal of Medical Internet Research.

[CR53] Yeager DS, Dahl RE, Dweck CS (2018). Why interventions to influence adolescent behavior often fail but could succeed. Perspectives on Psychological Science.

